# Measuring Functional Dependency Among Older Adults: Validation of the Physical Ability Questionnaire in ARIC Study

**DOI:** 10.1093/geroni/igaf122.2028

**Published:** 2025-12-31

**Authors:** Wuyang Zhang, James Pike, Pablo Martinez-Amezcua, Shoshana Ballew, Katherine Ornstein, Martha Abshire Saylor, Erin Kent, Anna Kucharska-Newton

**Affiliations:** Johns Hopkins Bloomberg School of Public Health, Baltimore, Maryland, United States; New York University, New York, New York, United States; Johns Hopkins Bloomberg School of Public Health, Baltimore, Maryland, United States; New York University, New York, New York, United States; Johns Hopkins University, Baltimore, Maryland, United States; Johns Hopkins School of Nursing, Baltimore, Maryland, United States; University of North Carolina at Chapel Hill, Chapel Hill, North Carolina, United States; The University of North Carolina at Chapel Hill, Chapel Hill, North Carolina, United States

## Abstract

Functional independence is critical to the wellbeing of older adults. We examined the psychometric properties of the Physical Ability Questionnaire (PAQ) in the Atherosclerosis Risk in Communities (ARIC) Study, an ongoing population-based cohort. We derived and validated a functional dependency score based on the Disablement Process Framework. The study population consisted of ARIC participants (N = 3,492; median age: 78 [range: 71-94]; 58% female; 22% Black) with complete PAQ assessment at Visit 6 (2016-2017). We conducted exploratory factor analyses of the 17 PAQ items and identified a one-factor model to derive a score of functional dependency. The construct validity of this score was examined against physical function (Short Physical Performance Battery), physical activity (moderate-to-vigorous, Baecke questionnaire), Fried frailty phenotype, all-cause hospitalization risk, and all-cause mortality using multivariable generalized linear regression and Cox proportional hazard models. The derived factor score exhibited moderately high internal consistency (Cronbach’s α = 0.85) and test-retest reliability (Intraclass Correlation Coefficient=0.69), along with moderate-to-strong cross-sectional associations with constructs of physical function (β=-1.57; 95% CI: -1.68, -1.47), physical activity (β=-56.08; 95% CI: -65.11, -47.05), and frailty (robust-to-prefrail: RRR=0.50; 95% CI: 0.45, 0.57; frail-to-prefrail: RRR=4.09; 95% CI: 3.27, 5.11). During a 5-year follow-up period, a one-unit increase in the functional dependency score was associated with elevated risks of hospitalization (HR = 1.43; 95% CI: 1.34, 1.52) and all-cause mortality (HR = 1.79; 95% CI: 1.59, 2.01). The derived functional dependency score can facilitate future research on aging-related outcomes in the ARIC cohort, and may serve as a basis for harmonization of functional assessment across studies.

